# Mesenteric fibromatosis with intestinal involvement mimicking a gastrointestinal stromal tumour

**DOI:** 10.2478/v10019-010-0051-7

**Published:** 2010-11-25

**Authors:** Marek Wronski, Bogna Ziarkiewicz-Wroblewska, Maciej Slodkowski, Wlodzimierz Cebulski, Barbara Gornicka, Ireneusz W. Krasnodebski

**Affiliations:** 1 Department of General, Gastroenterological and Oncological Surgery, Medical University of Warsaw, Warsaw, Poland; 2 Department of Pathology, Medical University of Warsaw, Warsaw, Poland

**Keywords:** mesenteric fibromatosis, desmoid tumour, gastrointestinal stromal tumour, GIST, differential diagnosis

## Abstract

**Introduction:**

Mesenteric fibromatosis or intra-abdominal desmoid tumour is a rare proliferative disease affecting the mesentery. It is a locally aggressive tumour that lacks metastatic potential, but the local recurrence is common. Mesenteric fibromatosis with the intestinal involvement can be easily confused with other primary gastrointestinal tumours, especially with that of the mesenchymal origin.

**Case report:**

We report a case of a 44-year-old female who presented with an abdominal mass that radiologically and pathologically mimicked a gastrointestinal stromal tumour.

**Conclusions:**

The diagnosis of mesenteric fibromatosis should always be considered in the case of mesenchymal tumours apparently originating from the bowel wall that diffusely infiltrate the mesentery.

## Introduction

Mesenteric fibromatosis (MF) or intra-abdominal desmoid tumour is a rare proliferative disease affecting the mesentery. MF is a locally aggressive tumour that lacks metastatic potential, but the local recurrence is common. It resembles gastrointestinal stromal tumours (GIST) that are mesenchymal neoplasms of the digestive tract and show a varied malignant potential. Although GISTs and mesenteric fibromatosis are distinct entities, they are often confused clinically, radiologically and not uncommonly pathologically as well. Misdiagnosis might result in inappropriate therapeutic decisions and worse prognosis.

We report a case of a 44-year-old female who presented with an abdominal mass that initially suggested a gastrointestinal stromal tumour.

## Case report

A 44-year-old female was admitted to our department because of the epigastric pain for the preceding two weeks. Her medical history was significant for arterial hypertension, Hashimoto thyroiditis and hypercholesterolemia. Her surgical history revealed a cesarean section. There were no desmoid tumours in her family.

**FIGURE 1.**
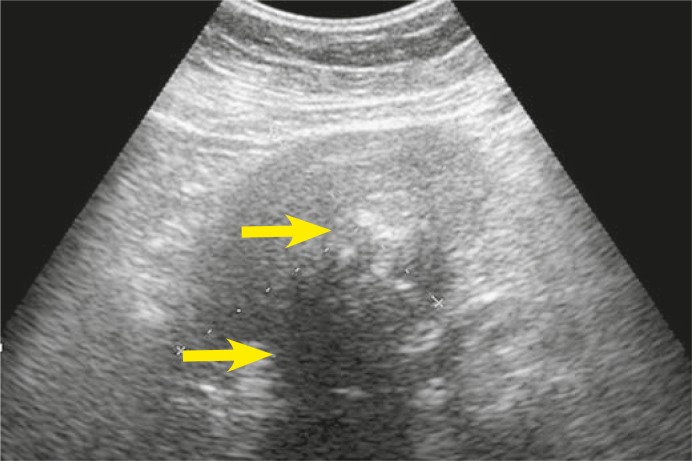
The sonographic appearance of an intra-abdominal desmoid with involvement of the small bowel: a well-defined grossly homogenous hypoechoic mass circumferentially encroaching the intestinal wall; the hyperechoic central part of the tumour corresponds to intraluminal air that results in posterior acoustic shadowing (arrows).

**FIGURE 2.**
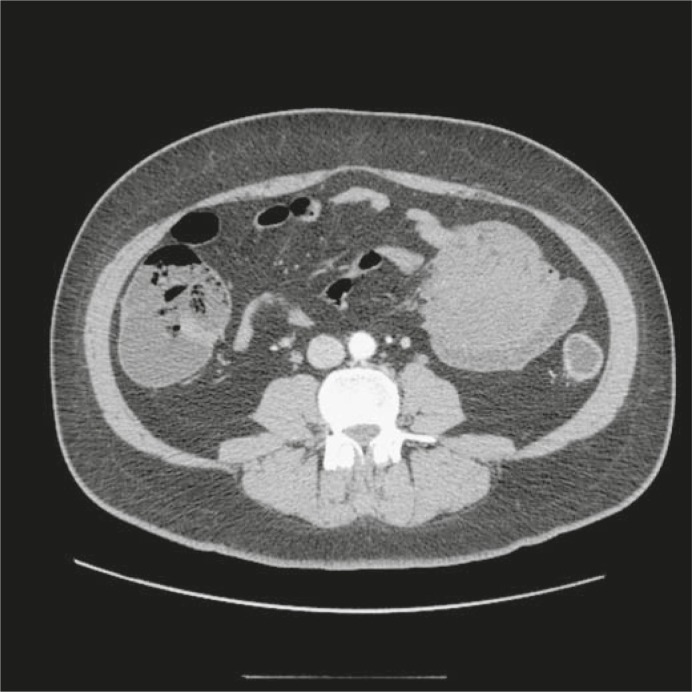
CT scan shows the desmoid tumour of the mesentery infiltrating the small bowel: a well-defined hypodense and homogenous mass diffusely attached to the bowel wall.

The physical examination revealed a mass on palpation in the mid-abdomen that was easily movable. The physical examination was otherwise normal. Laboratory findings were unremarkable. The level of CEA was within normal limits. A transabdominal ultrasound (US) showed an ovoid well-delineated homogenously hypoechoic mass that was 10.1 × 6.0 × 7.2 cm in size. There was a hyperechoic area in the central part of the tumour with posterior acoustic shadowing that corresponded to intraluminal air. The tumour was circumferentially attached to the wall of the small bowel (Figure 1). An abdominal computed tomography (CT) revealed a 8.2 × 7.2 × 7.4 cm mass infiltrating the small bowel. The tumour attenuation was of 33 Hounsfield units and it enhanced poorly and homogenously with an intravenous contrast (Figure 2). The above preoperative imaging studies suggested a GIST involving the small bowel.

**FIGURE 3.**
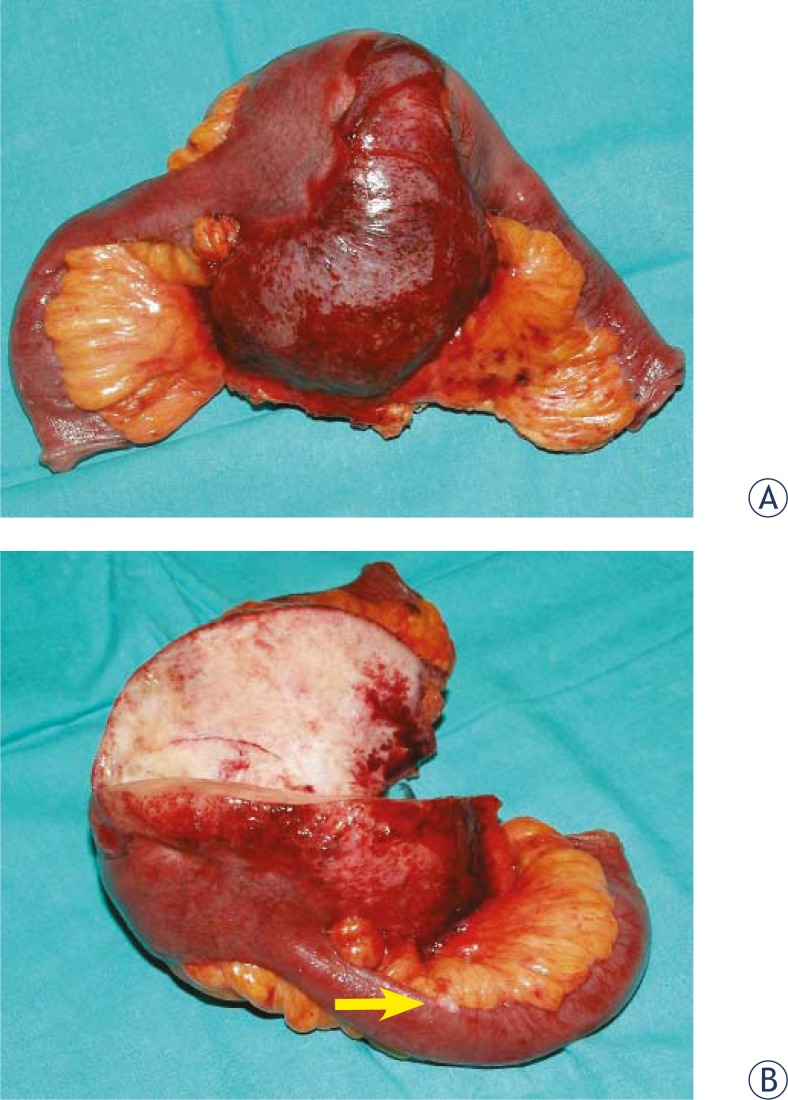
Macroscopic view of the resected specimen: A - a mesenteric mass encroaching the bowell wall, B – cut surface of the desmoid tumour showing grayish and glistening, homogenous desmoid tumour. A tubercule attached to the bowel serosa and mimicking peritoneal tumour deposits proved to be a mesothelial cyst (arrow).

The patient underwent an elective laparotomy. Intraoperatively, there was an approximately 10 cm well-circumscribed mass in the mesentery that infiltrated the wall of the small bowel and narrowed its lumen (Figure 3). On inspection, there were also 2–3 small tubercules attached to the serosa of the adjacent bowel that were included within the resection margins. Several similar lesions were found along the distal part of the small bowel and one of them was excised for the pathological evaluation. This gross appearance suggested a gastrointestinal stromal tumour with the peritoneal dissemination. The resection of a 25 cm segment of the small bowel was performed. The postoperative course was uneventful and the patient was discharged in a good health condition. A follow-up US revealed no desmoid recurrence a year after the operation.

**FIGURE 4.**
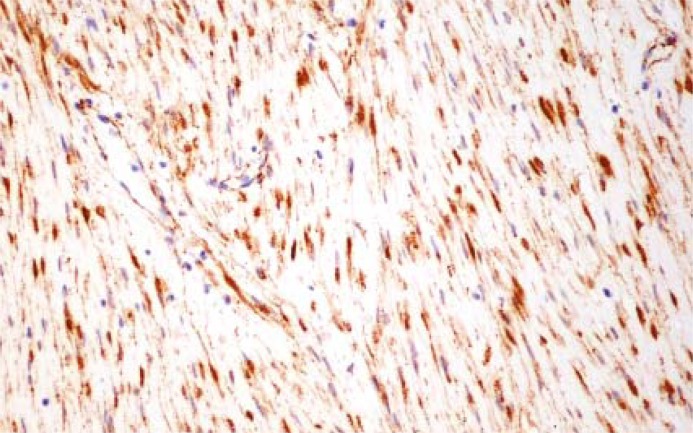
Microscopic view of mesenteric fibromatosis: immunostaining for beta-catenin.

The primary pathological diagnosis in this particular case was a CD117-negative gastrointestinal stromal tumour. The small serosal tubercules were found to be mesothelial cysts. The principal diagnosis was changed, however, after a consultation at a referral oncological centre. The microscopic examination of the resected specimen identified a fibromatosis in the mesentery. Histologically, the desmoid tumour was composed of spindle cells with elongated coma-shaped nuclei and the immunohistochemistry was negative for both CD117 and for CD34. Beta-catenin overexpression was present on immunohistochemistry (Figure 4). No mitoses were found in 50 high power fields.

## Discussion

Mesenteric fibromatosis is a type of fibroblastic proliferation affecting the mesentery that develops usually as a consequence of surgical trauma, but it may occur spontaneously. Patients with familial adenomatous polyposis (FAP, Gardner’s syndrome) are especially predisposed to the development of mesenteric fibromatosis.^1^ Desmoids develop in approximately 10% of FAP patients and most are intra-abdominal. Similarily, fibromatoses associated with FAP follow a more aggressive course and the recurrence after the resection is common.^2^

The pathogenesis of fibromatoses has been unclear for many years. Currently, these tumours are regarded as a clonal proliferation of myofibroblasts that show APC (adenomatous polyposis coli) gene mutations. These mutations lead to the over expression of beta-catenin.^1^,^3^,^4^

The clinical behaviour and the natural course of mesenteric fibromatoses are unpredictable. Some desmoid tumours remain stable for years and several cases of the spontaneous tumour regression without any treatment have been reported.^5^ Nevertheless, a progressive and invading tumour can result in a diffuse infiltration of the mesentery and bowel leading to intestinal ischemia or to the obstruction. The treatment modality in mesenteric fibromatoses is still controversial. The results of the treatment might be biased due to the unpredictable course of this disease with some tumours regressing or remaining stable without any treatment. The management of desmoids should be individualized and multimodal. The surgical resection should be performed only in localized tumours that do not invade the root of the mesentery. Intra-abdominal desmoids can be resected in 53–67% of cases.^6^ The aggressive surgical treatment of mesenteric desmoids may result in short bowel syndrome or multiple enterocutaneous fistulas requiring a long-term parenteral nutrition. Fibromatoses are locally aggressive tumours that tend to recur when incompletely resected. A high rate of recurrence after the surgical resection results from the incomplete resection, multicentric disease or surgical trauma as a new precipitating factor. Recent studies report comparable recurrence rates after R0 and R1 resection in extra-abdominal desmoids.^7^,^8^ Nevertheless, there are no data to support a similar influence of microscopically positive margins on recurrence in intra-abdominal desmoids.

Advanced and unresectable tumours or when the resection will result in short bowel syndrome are best treated within a clinical trial of cytotoxic chemotherapy or other experimental therapies. Radiotherapy is rarely used in intra-abdominal desmoids because of a high risk of radiation enteritis. There are no established chemotherapy regimens used in fibromatoses. Most chemotherapeutic protocols use doxorubicin.^9^ Recently, a successful therapy of a desmoid tumour resistant to traditional chemotherapeutic regimens was reported with imatinib, a tyrosine kinase inhibitor that is successfully used in advanced gastrointestinal stromal tumours.^10^,^11^

In a series reported by Bertagnolli *et al.*^9^, 96% of patients with mesenteric desmoids had either a radiographically stable disease or no recurrence for a median of 50 months using a multimodal treatment combining watchful waiting, surgery and chemotherapy.

The growth of desmoid tumours is usually limited to the mesentery, but the infiltration of the adjacent bowel is not uncommon. MF may infiltrate the muscularis propria or even the submucosa.^12^ The diagnosis of mesenteric fibromatosis is usually straightforward in the cases without a concomitant intestinal involvement. On the other hand, desmoid tumours encroaching the bowel wall present a diagnostic challenge and might be easily confused with primary intestinal tumours, especially gastrointestinal stromal tumours. Difficulties in differentiating these two tumours of distinct pathogenesis, natural course and prognosis are still not uncommon.^12^–^14^

Desmoid tumours are firm masses. On cross section these tumours are grayish and grossly homogenous. In comparison, GISTs are usually soft and fleshy. On cut surface, these tumours commonly have areas of necrosis and haemorrhage. In contrast to MF, the gross appearance of GISTs is highly dependant on their size, with large tumours being morphologically more heterogeneous. It follows that a large tumour that is firm and homogenous on cross section without obvious haemorrhagic and necrotic areas is highly suggestive of intra-abdominal fibromatosis. Sometimes, differentiating these two distinct tumours is difficult because of a possible clinical, macroscopic and even histological overlap. Nevertheless, the diagnosis of mesenteric fibromatosis is based on the microscopic examination and immunostaining. It is noteworthy that a CD117 antigen, expressed commonly in GISTs, can be positive in up to 75% cases of mesenteric fibromatosis.^11^–^13^ Moreover, both MF and GISTs express vimentin and stain variably for smooth muscle actin and desmin. In contrast to GISTs, MF does not express CD34 and S100 protein. Recently, the expression of beta-catenin was revealed in fibromatoses that might prove helpful in the differential diagnosis in doubtful cases.^1^,^15^,^16^

Radiographically, mesenteric fibromatosis may present as a mass-like or infiltrative lesion.^17^ Infiltrative desmoid tumours image as an ill-defined whorled thickening of the mesentery and are usually easily recognized. Mass-like desmoids are more challenging. These desmoids appear as well-defined tumours and are often confused with other primary neoplasms, especially gastrointestinal stromal tumours. Nevertheless, the distinction between these two tumours is important because of vital prognostic and therapeutic implications. In Table 1 the principal features of the mass-like mesenteric fibromatosis and gastrointestinal stromal tumours that might prove helpful in differentiating these two entities are summarized.^12^,^13^,^17^–^21^ On the other hand, it should be remembered that a recurrent mass after oncological operations may prove to be an intra-abdominal desmoid, and not necessarily a metastasis. Lee *et al.*^22^ reported a case of intraabdominal fibromatosis that occurred after the resection of a gastric stromal tumour. A similar case of intra-abdominal fibromatosis suspicious of local recurrence was reported after gastrectomy for gastric cancer.^23^ Failure to differentiate mesenteric fibromatosis from other tumours may lead to an inappropriate treatment and a worse prognosis.

In conclusion, the diagnosis of mesenteric fibromatosis should always be considered in mesenchymal tumours originating from the bowel wall that diffusely infiltrate the mesentery.

## Figures and Tables

**TABLE 1.** The clinicopathological features useful in differentiating mesenteric fibromatosis from gastrointestinal stromal tumours (GIST)

	**Mesenteric fibromatosis**	**GIST**
**Demographics**	25–35 year, F>M	50–60 year, F=M
**Clinical manifestations**	Asymptomatic, unless large, infiltrating bowel or compressing the ureters and vasculature; Common: abdominal pain Rare: GI bleeding, perforation	Common: abdominal pain, GI bleeding Rare: GI perforation, obstruction
**Location**	Small bowel mesentery	Anywhere along the GI tract; most common in the stomach and small bowel
**US**	Smooth well-defined margins, homogenous or heterogenous tumour of variable echogenicity	Extraluminal hypoechoic mass, small tumours are homogenous, large tumours are heterogenous with multiple anechoic patchy spaces or large central area of low echogenicity
**CT**	Well-defined homogenous mass, isodense or hyperdense relative to muscle, 1/3 show infiltrative margins, cystic degeneration is rare	Well-defined heterogenous mass with peripheral solid rim enhancing with contrast, central fluid attenuation (necrosis, haemorrhage, cystic degeneration); small tumours show homogenous enhancement
**MRI**	Tumour of low signal intensity relative to muscle on T1-weighted images and variable signal intensity on T2-weighted images	Tumour of low signal intensity relative to muscle on T1-weighted images and high signal intensity on T2-weighted images
**Gross appearance**	Hard and firm mass, cut with gritty sensation, white-greyish and glistening on cut section	Soft and fleshy tumours that often show areas of necrosis, haemorrhage and cystic degeneration on cut surface
**Microscopic view**	Homogenously distributed spindle cells without atypia, abundant collagen, thick-walled arteries and dilated thin-walled veins, mild cellularity, infiltrative pattern of growth	Spindle or epithelioid cells forming fascicles and palisades often with atypia and atypical mitoses, moderate to high cellularity, necrosis often present, expanding pattern of growth
**Immunostaining profile**	β-catenin (+)	β-catenin (−)
	CD117 (+) in up to 75%	CD117 (+) in 90%
	CD34 (−)	CD34 (+) in 42%
	vimentin (+)	vimentin (+)
	smooth muscle actin (+) in 75%	smooth muscle actin (+) in 63%
	desmin (+) in 50%	desmin (+) in 8%

GI, gastrointestinal
